# Review of current and future therapeutics in ABPA

**DOI:** 10.1177/20406223211047003

**Published:** 2021-10-23

**Authors:** Elisa Lewington-Gower, Ley Chan, Anand Shah

**Affiliations:** Department of Respiratory Medicine, Royal Brompton and Harefield Hospitals, Guy’s and St Thomas’ NHS Foundation Trust, London, UK; Department of Respiratory Medicine, Royal Brompton and Harefield Hospitals, Guy’s and St Thomas’ NHS Foundation Trust, London, UK; Department of Respiratory Medicine, Royal Brompton and Harefield Hospitals, Guy’s and St Thomas’ NHS Foundation Trust, London SW3 6NP, UK; MRC Centre for Global Infectious Disease Analysis, Department of Infectious Disease Epidemiology, School of Public Health, Imperial College London, London, UK

**Keywords:** ABPA, antifungal, *Aspergillus*, asthma, cystic fibrosis

## Abstract

Allergic bronchopulmonary aspergillosis is an allergic pulmonary condition caused by hypersensitivity to antigens of *Aspergillus sp.* found most commonly in patients with underlying asthma or cystic fibrosis. Host factors which alter the innate and adaptive immune responses to this abundant airborne fungus contribute to the development of chronic airway inflammation, bronchiectasis, and fibrosis. Traditionally, treatment has focussed on reducing fungal burden and immune response to fungal antigens. However, a significant proportion of patients continue to suffer recurrent exacerbations with progressive lung damage, and the side effect burden of existing treatments is high. New treatments including novel antifungal agents, monoclonal antibodies against aspects of the adaptive immune response as well as targeted immunotherapies may be better tolerated and achieve improved outcomes but have not yet been studied in large-scale randomised control trials.

## Introduction

Allergic bronchopulmonary aspergillosis (ABPA) is defined as an allergic pulmonary condition caused by hypersensitivity to allergens of the common saprophytic filamentous fungi *Aspergillus*. The most common pathogen in this genus, *Aspergillus fumigatus*, is abundant in both indoor and outdoor environments and does not usually cause disease in immunocompetent individuals.^1,2^ In immunocompromised individuals or in those with underlying lung disease however, it can cause a spectrum of disorders ranging from invasive aspergillosis to allergic aspergillosis.^3^ Allergic aspergillosis has been categorised into ABPA and severe asthma with fungal sensitisation.^4^

ABPA was first described by Hinson *et al.*^5^ in 1952 and is found in patients with asthma and cystic fibrosis (CF), as well as in rarer conditions, such as chronic granulomatous disease and hyper-immunoglobulin E syndrome. Characteristically, individuals with ABPA present with respiratory symptoms including poorly controlled asthma, wheeze, haemoptysis, and productive cough as well as systemic symptoms, such as fever and weight loss and can suffer recurrent exacerbations.^6^ In CF patients, ABPA may present with worsening symptoms or lung function that is not responsive to usual antibiotic treatments.^7^ ABPA can typically be a cause of large airway collapse and lead to bronchiectasis. In certain cases, ABPA may develop into chronic pulmonary aspergillosis. End-stage disease is typified by cor pulmonale and type-2 respiratory failure.^8^

The estimated prevalence of ABPA in asthma depending on definition criteria is between 0.7% and 22%, and therefore is likely to affect 4.8 million patients globally.^9^ The prevalence in severe asthma is likely to be much higher, for example, a prospective study of patients with severe asthma attending a tertiary centre clinic in Northern India showed a prevalence of 70%.^10^ The prevalence of ABPA complicating asthma in the United Kingdom is predicted to be between 50,000 and 250,000.^11^ Studies on CF have found a wide prevalence range between 2% and 19% dependent on definition criteria.^7,12,13^

In this review, we describe the diagnostic criteria and pathophysiology of ABPA, current therapeutic options, and future potential therapeutic targets.

## Diagnosis

The diagnosis of ABPA is based on a combination of clinical, serological, and radiological features. There are several criteria proposed for diagnosis including the Rosenberg–Patterson criteria,^14^ the ISHAM criteria (International Society of Human and Animal Mycology),^15^ and one proposed more recently by Asano *et al.*,^16^ as well as a modified ISHAM criteria based on latent class analysis proposed by Saxena *et al.*^17^ There are different criteria used for patients with CF (proposed by the CF Foundation) due to overlapping clinical features with bacterial exacerbations in CF,^7^ and for patients with allergic bronchopulmonary mycosis (ABPM).^15,16^ Table 1 shows a comparison of these diagnostic criteria.

**Table 1.** Comparison of diagnostic criteria for ABPA.

Rosenberg–Patterson criteria	ISHAM consensus criteria	Asano criteria	Saxena latent class analysis criteria	CF Trust criteria
Major criteria • Asthma • Presence of fleeting or fixed pulmonary opacities on chest radiograph • Immediate cutaneous hypersensitivity reaction to *Aspergillus fumigatus (A. fumigatus)* • Total serum IgE > 1000 IU/mL • Precipitating antibodies against AF • Peripheral blood eosinophilia • Central/proximal bronchiectasis with normal tapering of distal bronchiMinor criteria • Golden brown sputum plugs in expectorant • Positive sputum culture for *Aspergillus* species • Late (arthus-type) skin sensitivity to *A. fumigatus*	Predisposing conditions Asthma or cystic fibrosisObligatory criteria (both should be present) • Type-I *Aspergillus* skin test positive (immediate cutaneous hypersensitivity to *Aspergillus* antigen) or elevated IgE levels against *A. fumigatus* • Elevated total IgE levels (>1000 IU/mL)Other criteria (at least two of three) • Presence of precipitating or IgG antibodies against *A. fumigatus* in serum • Radiographic pulmonary opacities consistent with ABPA • Total eosinophil count >500 cells/µL in steroid naive patients	Require six or more for diagnosis • Current or previous history of asthma or asthmatic symptoms • Peripheral blood eosinophilia (≧500 cells/mm3) • Elevated total serum IgE (≧417 IU/mL) • Immediate cutaneous hypersensitivity or specific IgE for filamentous fungi • Presence of precipitins or specific IgG for filamentous fungi • Filamentous fungal growth in sputum cultures or bronchial lavage fluid • Presence of fungal hyphae in bronchial mucus plugs • Central bronchiectasis on CT • Presence of mucus plugs in central bronchi based on CT/bronchoscopy or mucus plug expectoration history • High attenuation mucus in bronchi on CT	Presence of all of the following; • Asthma • *A. fumigatus*-specific IgE >0.35 KUA/L • Serum total IgE levels >500 IU/mLTwo of the following: • *A. fumigatus*-specific IgG >27 mgA/L • Bronchiectasis on CT chest • Eosinophil count >500 cells/mL	Classic criteria • Acute or subacute clinical deterioration not attributable to another aetiology • Serum total IgE >1000 IU/mL (2400 ng/mL) unless receiving systemic corticosteroids • Immediate cutaneous reactivity to *Aspergillus* (>3 mm) or serum IgE antibody to *A. fumigatus* • Precipitating antibodies to *A. fumigatus* or serum IgG antibody to *A. fumigatus* • New or recent abnormalities on chest radiography (infiltrates or mucus plugging) or chest CT (bronchiectasis) that have not cleared with antibiotics and standard physiotherapyMinimal diagnostic criteria • Acute or subacute clinical deterioration (cough, wheeze, exercise intolerance, exercise induced asthma, change in pulmonary function, or increased sputum production) not attributable to another aetiology • Serum total IgE >500 IU/mL. If ABPA is suspected and the total IgE level is 200–500 IU/mL then repeat in 1–3 months. If on steroids, repeat once steroid treatment discontinued • Immediate cutaneous reactivity to *Aspergillus* (prick test wheal >3 mm with surrounding erythema, off systemic antihistamines) or serum IgE antibody to A. fumigatus • One of the following: *A. fumigatus* • Precipitins to *A. fumigatus* or IgG antibody to *A. fumigatus* • New or recent abnormalities on chest radiography (infiltrates or mucus plugging) or chest CT (bronchiectasis) that have not cleared with antibiotics and standard physiotherapy

ABPA, allergic bronchopulmonary aspergillosis; CF, cystic fibrosis; CT, computed tomography; ISHAM, International Society of Human and Animal Mycology.

These criteria use the results of several investigations to aid diagnosis, including *Aspergillus* skin tests, sputum cultures, peripheral eosinophilia, total serum immunoglobulin E (total IgE), *A. fumigates*-specific IgE, *A. fumigates*-specific immunoglobulin G (IgG), and radiological findings.^15^ Various cut-off values for these investigations have been proposed as several of these investigations may be abnormal in other fungal or related diseases, and there can be difficulties with the effects of treatment on some parameters.^15^

Some of these investigations may also be used in determining exacerbations and monitoring treatment efficacy.^7,16–18^ Total serum IgE levels can be used to monitor treatment with reductions in IgE of 25–50% correlating with improved symptoms and radiological appearances and increasing levels (e.g. a doubling in IgE level) suggesting an impending exacerbation.^15^ In addition, lung function can be used to determine the severity of underlying lung disease and monitor response to treatment. Fixed airflow obstruction and reduced lung volumes can be found in progressive disease.^19^ Sputum cultures are not diagnostic as they may be positive in patients without ABPA who are colonised with *Aspergillus* but are important in determining azole resistance prior to treatment.^15^

Central bronchiectasis (defined as bronchiectasis confined to the medial two-thirds or medial half of the lung) is the defining radiological feature of ABPA and is part of the Rosenberg–Patterson and Asano diagnostic criteria.^14,16,20,21^ Mucus impaction is another common finding on computed tomography (CT) imaging and high attenuation mucus (defined as mucus which is visually denser than paraspinal skeletal muscle or 70 Hounsfield units) is a pathognomonic feature of ABPA.^22–25^

## Staging

ABPA was originally staged by Patterson *et al.*^26^ in 1982. This staging involved five categories: acute, remission, exacerbation, steroid dependent asthma, and fibrotic lung disease. The International Society of Human and Animal Mycology (ISHAM) group proposed a new staging system, with seven stages ranging from 0 (asymptomatic) to 6 (advanced ABPA) as described in Table 2.^15^

**Table 2.** Clinical staging as proposed by the ISHAM working group.

Stage	Definition	Features
0	Asymptomatic	Global Initiative for Asthma (GINA) definition of controlled asthma Meets diagnostic criteria for ABPA No previous diagnosis of ABPA
I	Acute	Uncontrolled asthma/constitutional symptoms Meets diagnostic criteria for ABPA No previous diagnosis of ABPA
Ia	With mucoid impaction	Meets all criteria with mucoid impaction on chest X-ray, CT, or bronchoscopy
Ib	Without mucoid impaction	Meets all criteria without mucoid impaction on chest X-ray, CT, or bronchoscopy
2	Response	Clinical improvement (resolution of constitutional symptoms and improvement in asthma control) Major radiological improvement IgE decline by ≧25% of baseline at 8 weeks
3	Exacerbation	Clinical or radiological deterioration with increase in IgE ≧50%
4	Remission	Sustained clinico-radiological improvement with IgE levels remaining below baseline or increase <50% for ≧6 months on or off therapy other than systemic steroids
5a	Treatment dependent ABPA	Relapse on ≧2 consecutive occasions within 6 months of stopped treatment or has worsening clinical, radiological, or immunological parameters on tapering oral steroids/azoles
5b	Glucocorticoid-dependent asthma	Patient requires oral or parenteral glucocorticoids for asthma control while activity of ABPA is controlled as reflected by IgE levels of chest radiograph
6	Advanced ABPA	Type-II respiratory failure and/or cor pulmonale with radiological evidence of fibrotic findings consistent with ABPA on CT chest after excluding reversible causes of acute respiratory failure

ABPA, allergic bronchopulmonary aspergillosis; CT, computed tomography; ISHAM, International Society of Human and Animal Mycology.

Radiological staging has also been proposed by the ISHAM group.^24,27^ This divides patients into four stages with differing radiological appearances. These include ABPA-S (serological ABPA) which includes all diagnostic features of ABPA but no abnormality on CT, ABPA-*B* (ABPA with bronchiectasis), ABPA-HAM (ABPA with high attenuation mucus), and ABPA-CPF (ABPA with chronic pleuropulmonary fibrosis) which includes other radiological features including pulmonary fibrosis, parenchymal scarring, fibrocavitatory lesions, aspergilloma, and pleural thickening without the presence of mucoid impaction or HAM. In a study of 234 patients which were categorised in this way, the immunological severity also increased between the stages.^28^ These studies show clearly that ABPA represents a heterogeneous group of endotypes which will likely be important in evaluating future novel therapeutic strategies.

There are limited studies on the long-term prognosis of ABPA. In a study following 120 patients over 4 years, 33% went into remission, 20% became steroid dependent, and 2% developed end-stage fibrotic lung disease.^8^ Treatment can be effective in maintaining lung function and lack of treatment or late diagnosis can lead to progressive and even fatal disease.^14,15,29,30^ Therefore, early diagnosis and treatment of ABPA is crucial in preventing the development of serious and potentially irreversible lung damage, such as bronchiectasis or fibrosis.

## Pathophysiology

*A. fumigatus* is the most common pathogen involved in ABPA but other species including *A. flavus*, *A. niger*, and *A. oryzae* may also be involved.^31^ Other fungal species including *Schizophyllum commune* may cause similar pathology to ABPA, and this disease is termed ABPM.^32,33^

*A. fumigatus* produces tiny (2–5 micrometre) spores, known as conidia, which are able to remain in the atmosphere for long periods of time and are therefore inhaled in large numbers.^4,34^ *A. fumigatus* is ubiquitous in the environment and does not cause disease in most individuals.^35^ It is clear therefore that disease is caused by host factors, including genetic predisposition, which affect the host response to *Aspergillus* by affecting innate and adaptive immune responses. It is these innate and adaptive immune responses that are the current targets for treatment of ABPA, as well as treatment to reduce fungal burden.

Innate immune responses include non-specific physical, chemical, and cellular responses to pathogens, and defects in these processes have been shown to be involved in the pathogenesis of ABPA. A key factor in the development of disease is the ability of *Aspergillus* conidia to swell and develop hyphae. Innate immune systems are involved in preventing this by acting to clear fungal spores from the airways.

The first innate barrier to infection is the airway epithelium. In larger airways, the epithelial layer contains goblet cells and ciliated cells. Goblet cells produce mucus which traps foreign bodies (including fungal spores), and ciliated cells move the mucus up the airways towards the mouth, where it can be coughed up or swallowed. This mucociliary escalator is impaired in individuals with CF or asthma^36^ and therefore results in defective clearing of *Aspergillus* spores from the airway, predisposing patients to ABPA. *Aspergillus* itself is also involved in reducing the efficacy of the mucociliary escalator. For example, *Aspergillus* produces metabolites such as gliotoxin which impair ciliary beating.^37^

The composition of mucous may also be a factor predisposing to ABPA. Polymorphisms in the Cystic Fibrosis Transmembrane Conductance Regulator (CFTR) gene, which regulates the flow of sodium and chloride ions across cell membranes, affect the viscosity of mucous and increase the risk of ABPA.^38^ CF patients are known to have an increased risk of ABPA, and evidence has been found that mutations in the CFTR gene (insufficient for a diagnosis of CF) are associated with ABPA in asthmatic patients. Evidence from pooled results from four studies showed an increased likelihood of encountering CFTR mutations in patients with ABPA.^38^ The viscosity of mucus is another target in the treatment of ABPA.

ABPA is characterised by eosinophilic recruitment to the airways and peripheral eosinophilia. Eosinophils have recently been shown to undergo a process of extracellular trap cell death in response to *Aspergillus*. Eosinophils undergo cytolysis and release filamentous chromatin fibres (eosinophilic extracellular traps, EETs).^39,40^ EETs act to immobilise *Aspergillus via* hydrophobic interactions; however, EETs may also contribute to increased mucus viscosity and sputum plug formation.^41^ A case report showed abundant EETs in mucous plugs found in a patient with ABPA, and a significant reduction in EETs once the patient was treated with steroids.^39^ In addition, there is evidence that EETs are not effective in killing *Aspergillus*, and so may contribute to pathogenesis.^42^

A further innate immune response occurs within the smaller airways, where Type-II pneumocytes are responsible for secreting surfactant proteins, including surfactant proteins A and D. In addition to their other functions, these act as opsonins that bind *Aspergillus* conidia and target them for phagocytosis by neutrophils and macrophages.^43,44^ Genetic polymorphisms that cause changes in the collagen region of surfactant protein A2 have been shown to be associated with increased levels of IgE and increased eosinophilia compared to patients with ABPA who lacked these single-nucleotide polymorphisms (SNPs), suggesting that changes in the function of surfactant proteins may contribute to worsened disease.^45^

A number of other innate cells including alveolar macrophages, neutrophils, monocytes, and dendritic cells are involved in *A. fumigatus* innate responses,^46,47^ which they detect *via* pattern recognition receptors (PRRs).^4^ These receptors, located on the cell wall detect pathogen-associated molecular patterns (PAMPs) including B glucan, chitin, and galactomannan.^4^ Toll-like receptors (TLRs) are located within the plasma membranes of cells and on intracellular endosomes and detect PAMPs on the fungal cell wall. Binding to these receptors, triggers secretion of pro-inflammatory cytokines, and TLR activation on dendritic cells causes propagation of an adaptive immune response.^48^ Genetic polymorphisms in TLR3 and TLR9 and mannose binding lectin have been shown to cause susceptibility to ABPA, and TLR agonists have been considered as adjuvants in allergy immunotherapy treatment including in ABPA.^48–51^

Innate lymphoid cells (ILCs) are a recently discovered group of innate immune cells, and group-2 ILCs (ILC2s) have been shown to have a significant role in allergic diseases including asthma.^52^ While there are no studies on ILC2s in ABPA specifically it is likely that these cells which respond to allergen exposure and when activated cause release of type-2 cytokines (including interleukin (IL)-5 and IL-13) resulting in eosinophilia and mucus hypersecretion may also be involved in the pathogenesis of ABPA and therefore may be a future therapeutic target. A recent study showed that in a murine model of eosinophilic asthma, inhibition of ILC2s by 2’3’-cGAMP resulted in decreased airway hyper-responsiveness and lung eosinophilia in response to challenge with *Aspergillus flavus*,^53^ suggesting that inhibition of ILC2s may reduce type-2 inflammation in patients with ABPA.

The adaptive host response involves T and B lymphocytes and is pathogen specific. In general, the host response to a pathogen may be either a T-helper (Th) 1 or Th2 response and both cell types produce specific cytokines. Th1 responses are generally pro-inflammatory and involve cytokines, such as interferon gamma (IFN γ), IL-2 and tumour necrosis factor beta (TNF β). Th1 cytokines enhance cytotoxic and macrophage activity and therefore promote fungal clearance.^54^ The host response to *Aspergillus* in ABPA however is skewed towards a Th2 response, and this is thought to cause progressive disease.^6^ Th2 responses counteract Th1 responses and promote IgE and eosinophilic responses. Th2 cytokines include IL-4, IL-5, IL-10, and IL-13. IL-4 stimulates activated B cells and promotes differentiation of B cells into IgE producing plasma cells.^6^ IL-5 is a mediator for eosinophil production and activation. IL-13 induces airway hyper-responsiveness, goblet cell metaplasia, and mucous hypersecretion.^55^ SNPs in IL-4R and IL-13 genes have been shown to be associated with increased risk of ABPA^56^ and the 1082GG genotype of the IL-10 promoter has been associated with the development of ABPA in CF patients.^57,58^ Monoclonal antibodies have been designed to target aspects of the Th2 inflammatory response and have been shown to be effective in the treatment of ABPA.^59–62^

As well as affecting the viscosity of mucus, mutations in the CFTR gene have also been shown to affect the adaptive immune response. For example, mutations in the CFTR gene have been shown to be involved in maintaining the balance between Th1 and Th2 responses, with CD4+ T-cells derived from CF patients exhibiting Th2 bias *in vitro* compared with controls.^63^ *A. fumigatus* has also been shown to generate extensive lung inflammation and an enhanced Th2-biased immune response in CFTR deficient mice compared with controls.^64^

Observational studies have shown an increased risk of ABPA in patients who express HLA-DR2 and/or DR5 but not HLA-DQ2. HLA-DRB1*1501 and 1503 are associated with higher risk and HLA-DQB1*0201 are associated with lower risk.^65–67^ It is thought that these patients express a major histocompatibility complex/HLA that restrict the phenotype of expression in antigen presenting cells and skews the immune response towards a Th2 response.^6^

*A. fumigatus* also has various mechanisms which drive the adaptive immune response towards a Th2 response rather than a Th1 response. For example, *A. fumigatus* has been shown to inhibit IFNβ signalling through the JAK-STAT pathway, which leads to a reduction in the chemokine CXCL 10 and drives the response towards a Th2 response. *Aspergillus* has also been shown to cause activation of certain receptors (protease-dependent receptor 2 and tyrosine-protein phosphate non-receptor type) in bronchial epithelial cells which results in suppression of CXCL 10, promoting a Th2 response,^68,69^ in addition to secretion of proteases such as alkaline serine proteases which have elastolytic and collagenolytic activity and cause damage to epithelial cells and airway structural components.^70–72^ Repair mechanisms in response to damage by *Aspergillus* proteases, mast cell degranulation, eosinophils, and EETs result in proliferation of epithelial cells, endothelial smooth muscle cells and fibroblasts, resulting in remodelling of the airways, and development of bronchiectasis.^41,73–75^ Due to these factors, antifungal therapeutics aiming to reduce fungal colonisation of the airways are an important consideration in management of ABPA.

## Current and future therapies

**Figure 1.**
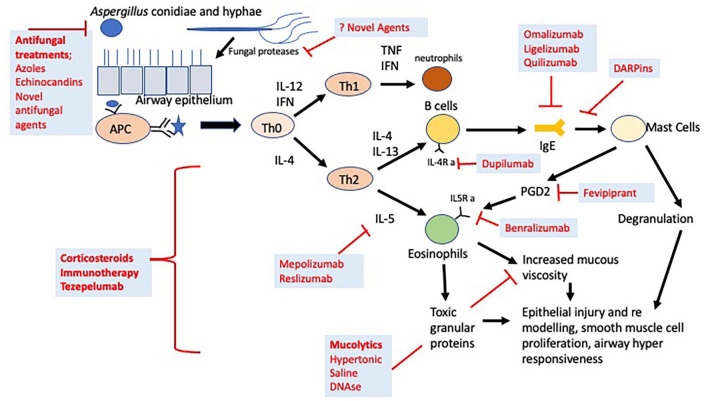
Summary of action of different therapeutic agents in ABPA.

The overall therapeutic goal of ABPA management is to minimise the pro-inflammatory response and reduce airway fungal burden to control symptoms, reduce exacerbations, maintain and normalise lung function, and prevent radiological progression.^4,27^ Treatment therefore has traditionally included immune suppression using steroids and antifungal therapy to reduce fungal burden. Novel treatments including monoclonal antibodies, immunotherapy, and novel antifungal agents may be of benefit in the treatment of ABPA but have not been studied in large-scale randomised control trials. A summary of the action of different therapeutic agents in the pathogenesis of ABPA is shown in Figure 1.

At present, glucocorticoids are the current mainstay of treatment; however, there is a paucity of clinical trials evaluating effectiveness, dose, and duration. To date, there have been two dose regimens for oral glucocorticoid therapy studied in the literature: medium and high dose.^76^ The medium-dose regimen consisted of prednisolone 0.5 mg/kg/day for 1–2 weeks, then alternate day therapy for 6–8 weeks, and finally tapering by 5–10 mg every 2 weeks until ceasing after 3–5 months. The high-dose regimen consisted of prednisolone 0.75 mg/kg/day for 6 weeks followed by 0.5 mg/kg/day for 6 weeks. The dose was then reduced by 5 mg every 6 weeks to complete a treatment duration of 6–12 months.^76^ A retrospective study in 55 patients with ABPA-S showed that treatment with high-dose steroid regime induced remission (defined as a reduction in serum IgE levels by at least 25%) in all patients at 3 months.^28^ This indicates that the use of systemic corticosteroids may be significant in reducing progression to radiologically apparent disease; however, the study has a number of limitations including size, its retrospective nature and the lack of a control group.

A randomised control trial of 92 patients with asthma found that the medium dose regimen was as effective and less harmful than high-dose regimen in acute ABPA, with no significant difference in time to exacerbation or progression to steroid dependent ABPA. There was in addition a significantly higher number of patients with steroid-related complications in the higher dose group.^76^ A recent review by Ishiguro *et al.*^77^ into patients with ABPM showed that systemic corticosteroids are also effective in this related condition. Intravenous pulsed steroids have been studied and shown to be beneficial in children with refractory ABPA exacerbations associated with long-term steroid use.^78,79^ Although effective, the use of systemic glucocorticoids is limited by significant side effects including obesity, osteopenia, development of type-2 diabetes, insomnia as well as many other effects.^80^ In addition, long-term glucocorticoid use can cause downregulation of glucocorticoid receptors inducing a steroid-resistant state.^80^

Inhaled steroids have been investigated in numerous case studies for treatment of ABPA. These studies often included patients on systemic corticosteroids in addition and assessed efficacy in a variety of different ways including radiologically and with lung function, rather than serology.^81,82^ The recommendation of the ISHAM committee was that high-dose inhaled corticosteroids should not be used in isolation in ABPA due to lack of evidence of efficacy.^15,83,84^

Despite corticosteroids being the mainstay of treatment in ABPA, 50% of patients relapse once systemic steroids are reduced and up to 45% become steroid dependent.^15,83^ Given the significant side effect profile of steroid treatment often antifungal strategies are considered in steroid dependency.

## Antifungal therapies

As detailed above, antifungal agents have been used in ABPA as it is thought reducing the fungal burden in the airways will reduce the antigenic stimulus and therefore reduce inflammation. The easiest way to reduce fungal burden is to reduce *Aspergillus* spore exposure. Patients may therefore be advised to avoid high-risk environments, for example, areas with decomposing matter and mouldy indoor environments.^4^ Given the ubiquity of *Aspergillus* in the environment however, this is unlikely to reduce the fungal burden significantly for most patients. Other strategies to reduce fungal burden include treatment with antifungal agents.

Oral triazole antifungal drugs are effective against *A. fumigatus* and are predominantly first-line therapy in the management of *Aspergillus*-related infection and allergy in chronic respiratory disease. Triazole antifungals include first-generation drugs, including itraconazole and fluconazole and second-generation triazole antifungals, such as voriconazole, posaconazole, and isavuconazole.^85^ Ketoconazole was the first antifungal agent used to treat ABPA; however, this has now been largely superseded by itraconazole.^86^ In a study comparing itraconazole to placebo in steroid-dependent ABPA, it was shown that itraconazole reduced steroid dose by more than 50% and reduced total IgE levels by more than 25%. There were also clinical improvements including improved exercise capacity, resolution of radiological opacities on chest radiograph and improved spirometry, although these were not statistically significant.^87^ A further study in clinically stable patients showed a reduction in sputum inflammatory markers, serum total IgE levels, and frequency of exacerbations with itraconazole.^88^

A study comparing the effectiveness of steroid monotherapy *versus* itraconazole monotherapy in acute ABPA showed no difference in the proportion of patients achieving full remission after 3 and 6 months of treatment and no difference in time to first exacerbation, which the authors suggest shows itraconazole can be used as a steroid alternative.^89^ However, their findings did show that steroid treatment resulted in a significantly greater initial response (within 6 weeks), measured as a composite score consisting of improved symptoms, improved chest radiograph appearances and decline in serum IgE by more than 25%. The rate of adverse outcomes however, including cushingoid habitus, weight gain and acne was significantly higher in the steroid group. A limitation of this study was that there was a significant proportion of patients (12%) who did not initially respond to itraconazole and were excluded from subsequent follow-up investigations, with no ability to predict non-response.

Long-term azole use, however, can have several adverse effects including gastrointestinal (GI) disturbance, hyperlipidaemia, peripheral oedema, peripheral neuropathy, and heart failure.^90^ Itraconazole is a cytochrome P450 inhibitor and therefore may have interactions with other medications including corticosteroids. Therefore, when used in cases of steroid dependency, effectiveness of azole therapy when given in combination with steroids may potentially relate to increased steroid bioavailability. The use of inhaled corticosteroids (e.g. fluticasone and budesonide) can result in adrenal suppression and Cushing’s syndrome.^91^

Alternative newer azole medications (e.g. posaconazole and voriconazole) have been used in treatment failure or intolerance with itraconazole.^92^ Posaconazole and voriconazole have been shown to induce clinical response in 78% and 70% of patients, respectively, in a study of 25 patients with previous itraconazole treatment failure, with 75% of patients able to discontinue oral corticosteroids, a reduction in serology, healthcare utilisation, and short-term beta-agonist use and improvement in radiological appearances at 9 months.^93^ The study also noted significant adverse events with voriconazole (40%) with 26% of patients requiring treatment cessation, with no significant adverse effects with posaconazole.

In the CF population, there is some evidence to suggest that posaconazole may be more effective than other triazoles in the treatment of ABPA. A retrospective study of 32 CF patients with ABPA treated with itraconazole, voriconazole, and posaconazole showed a significant reduction in *Aspergillus* IgE with posaconazole in comparison to corticosteroids only or other azoles,^94^ with *Aspergillus* IgE levels inversely correlated to drug levels. The authors suggest the improved serological response to posaconazole may be due to its improved bioavailability and ability to achieve therapeutic drug levels or enhanced antifungal activity. The study has a number of limitations, however, and randomised controlled trials are required to look at the relative effectiveness of novel azole therapies in ABPA. There are a number of novel inhaled azole compounds in clinical trials with an aim to reduce the systemic side effect profile often related to azole therapy. Itraconazole has been developed as a dry powder inhaler and is currently in phase-II clinical trial.^95^ In addition, a novel triazole, PC945 has been shown to be more effective against *Aspergillus* than voriconazole *in vitro* and *in vivo*, has limited systemic bioavailability and is due to commence phase-III clinical trials in 2021.^96^

Other currently available classes of antifungal therapy include the echinocandin group (e.g. caspofungin, micafungin, and anidulafungin) and the polyenes (e.g. amphotericin). No trials as yet have been conducted into the use of echinocandins in the management of ABPA. However, they have been used in other *Aspergillus* diseases where azole resistance is an issue.^3^ Newer echinocandin agents are currently in clinical trial with modified pharmacokinetic properties allowing for reduced frequency administration (e.g. Rezafungin),^97^ with phase-3 trials in the setting of invasive fungal disease ongoing. Further novel echinocandin agents with oral bioavailability (e.g. ibrexafungerp) are also in phase-3 clinical trials.^98^ Whether these agents will be useful in ABPA management remains to be studied.

Amphotericin B in nebulised form has been used in the treatment of ABPA. Nebulised amphotericin (in sodium deoxycholate formulation) was found to be effective in 3 of 21 patients; however, the remaining 18 patients had to discontinue due to bronchospasm.^99^ It is thought that lipid formulations (e.g. ambisome) may be better tolerated and there is some evidence for its use in paediatric patients with CF.^100,101^

There are additionally several completely novel antifungal agents in late phase clinical trials that may potentially be of use in the treatment of ABPA. Olorofim inhibits the enzyme dihydroorotate which is a key step in pyrimidine synthesis. It has been shown to be active *in vitro* against *Aspergillus* and phase-2b clinical trials in the setting of invasive fungal infections are ongoing.^102^ Fosmanogepix is a pro-drug of manogepix, which is an inhibitor of the fungal enzyme GPI-anchored wall transfer protein 1 (Gwt1) which is involved in glycolipid biosynthesis and has been shown to have significant *in vivo* efficacy against *Aspergillus*. It is currently in phase-2 clinical trials and has shown excellent oral bioavailability, favourable drug–drug interaction and tolerability.^103^

*Aspergillus* additionally produces a variety of different toxins which affect the airway and interact with the host response and there has been interest in these as therapeutic targets in ABPA. Alkaline protease 1 (Alp1) may be an example of a potential new target for treatment in ABPA. This *A. fumigatus* serine protease has been shown to cause airway hyper-responsiveness, inflammation, and smooth muscle contraction which contributes to pathogenesis of ABPA and is independent of the host immune response.^104^ Targeting such secreted products may thus be a novel future approach to managing this complex disease.

## Monoclonal antibodies

There is increasing evidence for the use of monoclonal antibodies in treating ABPA. Omalizumab (Xolair) is a humanised monoclonal IgG antibody against IgE. It acts to bind free serum IgE and down regulates cell-surface high-affinity receptors for IgE (FcεR1) on basophils and mast cells.^60^ The dose is dependent on the initial IgE level (0.016 mg/kg/IU) with an upper IgE limit of 1500 IU/mL and a maximum dose of 1200 mg monthly. As serum IgE levels are usually very high in ABPA, large doses are usually required.^59^

A randomised cross-over study evaluated the effectiveness of omalizumab compared with placebo in 13 patients with ABPA and found that there was a significant reduction in exacerbations in the treatment arm and FENO reduced significantly from baseline. There were no adverse events.^60^ A systematic review which included 102 patients with predominantly prior treatment failure with steroids and antifungals from 30 studies was done to assess the effectiveness of omalizumab. This showed there was a reduction in clinical symptoms, exacerbation rates, steroid use, serum total IgE levels, and FENO following treatment with omalizumab. There was a suggestion that there were greater effects in patients with higher baseline total IgE levels receiving treatment via the subcutaneous route. However, the majority of the data in this study was derived from case reports.^59^ Omalizumab has also been reviewed in the CF population. A retrospective study into CF patients with ABPA showed no significant reduction in cumulative steroid dose, days on IV antibiotics or days in hospital.^105^ However, a small proportion (4/11 patients) managed to reduce their steroid dose by over 50%. All the studies performed to date in CF and non-CF ABPA are limited by a lack of placebo control, with small patient numbers and often retrospective analysis. Further prospective clinical trials are required to analyse the long-term effectiveness of omalizumab or other anti-IgE monoclonal antibodies (e.g. ligelizumab and quilizumab) in ABPA.^106^

Mepolizumab is a humanised monoclonal antibody to IL-5, which is a key mediator in eosinophil differentiation, activation, migration, and survival. A systematic review evaluated the effectiveness of mepolizumab in ABPA in seven studies, with a total of eight patients.^61^ Subcutaneous mepolizumab 100 mg every 4 weeks was given to all patients. Eosinophil numbers dropped significantly in all patients, but only four patients had a significant reduction in total IgE levels. There were however improvements in FEV1 and radiological findings, and there was a clinical improvement in all patients with no adverse effects identified. Reslizumab and benralizumab also interrupt signalling via IL-5. Reslizumab is a second anti-IL-5 monoclonal antibody and benralizumab is a monoclonal antibody against the α unit of the IL-5 receptor. Both have been shown to reduce blood eosinophil levels in asthma patients with potential efficacy in ABPA.^106^ A retrospective study of anti IL-5/5R treatment in patients with severe asthma with fungal sensitisation and ABPA showed a reduction in exacerbations in the ABPA subgroup.^107^ However, this study is limited by the small patient numbers in the ABPA subgroup (*N* = 9) and large randomised controlled trials are required to definitively understand the utility of anti-IL-5 therapy in ABPA.

Dupilumab is an IL4-Rα antibody that has been used in atopic dermatitis, severe asthma, and chronic rhinosinusitis and inhibits Th2 cytokine signalling via IL-4 and IL-13. A recent case report documented the use of dupilumab in a patient with ABPA who had previously failed treatment on itraconazole, omalizumab, and benralizumab.^62^ The patient was commenced on dupilumab in addition to high-dose inhaled corticosteroid, long acting beta-agonist and prednisolone 20 mg and symptoms resolved at 4 months. The patient was able to taper steroids successfully, and there was significant improvement in lung function sustained at 8 months. IgE levels and eosinophil levels had also normalised by 8 months. Dupilumab holds significant potential as a therapeutic in ABPA and a phase-III randomised control trial of dupilumab in asthma patients with ABPA is currently underway (NCT04442269).

Tezepelumab is a monoclonal antibody against thymal stromal lymphopoietin (TSLP) which acts as an upstream mediator of the inflammatory response to common asthma precipitants including viruses, allergens, and other airborne irritants. TSLP acts on multiple aspects of the inflammatory response including allergic inflammation, eosinophilic inflammation, neutrophilic inflammation, and airway remodelling. Tezepelumab has been shown to reduce exacerbation rates, improve lung function and reduce symptoms in patients with a wide variety of asthma phenotypes.^108^ Given its actions on Th2-mediated immune responses and airway remodelling it again may show significant promise as a future therapeutic agent in ABPA.

Similarly, Fevipiprant, an antagonist of prostaglandin D2 receptor 2 has been shown to reduce eosinophilic airway inflammation in a randomised controlled trial of moderate – severe asthma patients.^109^ This small trial showed a reduction in sputum eosinophil percentage by 4.5 times and may therefore be of benefit in patients with ABPA.

Future potential therapeutics targeting IgE-mediated Th2 inflammation includes designed ankyrin proteins (DARPins) which prevent IgE-mediated activation of effector cells. They act to disrupt the formation of IgE/receptor complexes and break down formed complexes.^110^ Several DARPins have been shown to be effective *in vitro* including DARPIN E2_79 and D11^111,112^. While the early results are promising and may be applicable for use in ABPA, further studies are required.

## Mucolytics

In addition to anti-inflammatory and antifungal drugs, treatments that reduce mucus viscosity have been used to reduce the symptom burden in patients with ABPA. Hypertonic saline can be used (following appropriate trial dose and in conjunction with salbutamol to prevent bronchospasm) to reduce sputum viscosity and promote clearance, although no studies on long-term benefit have been performed.^113^

There are a number of other licenced mucoltyics available including nebulised Dornase-alpha and *N*-acetylcysteine; however, no ABPA-specific clinical trials have been performed. Dornase-alpha is potentially of benefit in ABPA, given the significant contribution of filamentous chromatin-rich EETs to sputum viscosity. A recent observational study of DNase use in the UK CF population showed modest long-term improvements in patients with lower lung function (FEV1 < 70% predicted) but was not able to distinguish any additional benefit in the ABPA subset.^114^ A recent case series reviewed the use of bronchoscopy with instillation of DNase in five patients with CF and ABPA who developed lobar atelectasis, and reported full lung re-expansion in all cases.^115^ However, it is possible that bronchoscopy and manual removal of secretions resulted in this good outcome rather than DNase use and further randomised trials are needed to determine the efficacy of DNase in ABPA.

## Immunotherapy

Allergy immunotherapy has been shown to be effective in atopic asthma and allergic rhinitis and is effective in preventing new allergen sensitivities. Small amounts of allergen are given sublingually or subcutaneously with the aim of inducing immunological tolerance which is maintained even after discontinuation of treatment. The first effect is the desensitisation of FcεR1 bearing mast cells and basophils, and this is followed by T-cell tolerance which is mediated by IL-10 and TGF-β. Immunotherapy has been shown to decrease nasal symptoms in allergic rhinitis and prevent progression to asthma.^116^

Immunotherapy may in the future have a role in reducing the allergic response to *Aspergillus sp* seen in ABPA. However, this treatment has a significant side effect profile including risk of anaphylaxis, with a reported systemic reaction to subcutaneous allergy immunotherapy of 0.1% per injection. Patients have been shown to require treatment monthly for at least 4 years to induce long-term benefit once treatment is discontinued. Sublingual immunotherapy has a better safety profile with similar efficacy. However, both routes of administration have significant issues with adherence, given the need for repeated doses.^116^

TLR agonists have been investigated as adjuvant treatment in immunotherapy for common allergens involved in allergic rhinitis. They have been shown to favour an anti-allergic inflammatory profile favouring Th1 responses and when administered with allergens promote immunological tolerance.^48^ Several TLR agonists have been investigated in the context of allergy immunotherapy and have shown efficacy in enhancing Th1 responses, although these data are largely from *in vitro* and animal models. Synthetic TLR4 and TLR9 agonists have been evaluated in clinical trials and synthetic TLR2, TLR5, and TLR7 have shown efficacy in human *in vitro* studies and animal *in vivo* studies. Similarly, monophosphoryl lipid A is an adjuvant that binds TLR4 and short segments of DNA with CpG motifs bind TLR 9.^116^ It may be possible in the future therefore to use TLR agonists as adjuvants in allergen immunotherapy directed against *Aspergillus*.

## Therapy based on current available evidence

A suggested protocol for treatment of ABPA^15^ would be to initiate medium-dose steroid treatment or antifungals (itraconazole) in clinical stage 1, depending on patient factors and patient-specific risks for each treatment. Physiotherapy and mucolytic therapy as necessary should be instituted. Treatment response should be monitored initially at a 6- to 8-week interval with serum IgE levels, chest radiography, lung function, and quality-of-life questionnaires. The aim of therapy should be to reduce the IgE level by 25–50% and achieve clinical remission and stability. Steroids can then be reduced or tapered in remission (stage 2). A further exacerbation (stage 3) should be treated with either steroids alone or in combination with an antifungal (itraconazole, or if no improvement posaconazole or voriconazole). If the patient becomes treatment dependent (stage 5), alternative antifungals, pulsed methylprednisolone, nebulised amphotericin, or biologic agents could be considered.

## Conclusion

ABPA is caused by an abnormal host response to *Aspergillus sp*, with failure in innate and adaptive immune systems resulting in reduced clearance of fungal spores from the airways and the ability for *Aspergillus* to form hyphae. This results in further Th2-weighted immune responses which in combination with substances secreted by *Aspergillus* itself, such as fungal proteases lead to airway hyper-responsiveness and excess mucus production, airway remodelling, inflammation, bronchiectasis, and fibrosis.

The traditional treatments for ABPA focus on reducing the immune response with corticosteroids and reducing fungal burden with antifungal agents. However, despite this, a significant number of patients continue to suffer from active disease and exacerbations which cause further lung damage, bronchiectasis, and fibrosis. In addition, many of the current treatments have significant adverse effects which cause increased morbidity.

Targeted immune treatments against aspects of the aberrant Th2 response have been shown to be effective and novel antifungal agents may be better tolerated and should be considered in patients with hard to treat disease or recurrent exacerbations. However, despite the potential role of novel biologic and antifungal therapies there is a critical lack of large-scale randomised control trials in ABPA which is a widespread global disease with significant health burden and morbidity. The current evidence for novel biologic and antifungal therapies is limited to case series and subgroups of larger trials, limiting the therapeutic options for patients. An understanding of disease heterogeneity in ABPA and endotypes will also be critical to ensure therapeutic stratification and success of future clinical trials.
